# Enterovirus A71 Genogroups C and E in Children with Acute Flaccid Paralysis, West Africa

**DOI:** 10.3201/eid2204.151588

**Published:** 2016-04

**Authors:** Maria D. Fernandez-Garcia, Ousmane Kebe, Aichatou D. Fall, Hamet Dia, Ousmane M. Diop, Francis Delpeyroux, Kader Ndiaye

**Affiliations:** Institut Pasteur, Dakar, Senegal (M.D. Fernandez-Garcia, O. Kebe, A.D. Fall, H. Dia, K. Ndiaye);; World Health Organization, Geneva, Switzerland (O.M. Diop); Institut Pasteur, Paris, France (F. Delpeyroux);; Institut National de Santé et de La Recherche Médicale, Paris (F. Delpeyroux)

**Keywords:** enterovirus A71, subgenogroup C2, nonpolio enteroviruses, genogroup E, acute flaccid paralysis, viruses, West Africa

**To the Editor:** Human enterovirus 71 (EV-A71) of the species e*nterovirus A*, genus *Enterovirus*, and family *Picornaviridae* is a serious public health threat because it can cause large outbreaks of hand, foot and mouth disease (HFMD). In addition, EV-A71 has been associated with severe and sometimes fatal neurologic complications that affect mostly infants and children and that range from aseptic meningitis and encephalitis to poliomyelitis-like acute flaccid paralysis (AFP) (*1*). EV-A71 has been classified into 7 genogroups, A–G, on the basis of the diversity of the nucleotide sequences of the viral protein 1 (VP1) capsid (*1**,**2*). Since 1997, increasing epidemic activity of genogroups B and C has been reported in the Asia-Pacific region and has caused large HFMD outbreaks with high rates of illness and death. Subgenogroup C2 was identified as the main causal agent associated with cases of fatal encephalitis in the devastating HFMD outbreaks of Taiwan in 1998 and Australia in 1999 (*1*). Since those outbreaks, C2 has been frequently reported in countries in Asia, Europe, North America, and South America and has been associated with neurologic complications and fatal infection (*1*). Although genogroups E and F were recently discovered in Africa, the epidemiology of EV-A71 has been largely unexplored in this continent. Only 4 AFP cases associated with EV-A71 infection have been reported in Africa (*2**–**5*), and the only outbreak caused by EV-A71 occurred in Kenya in 2000 among a small number of HIV-infected orphans and was attributed to genogroup C (*6*).

To investigate the circulation and genetic diversity of EV-A71 in West Africa, we retrospectively analyzed 236 nonpolio enterovirus (NPEV) isolates obtained through routine poliomyelitis surveillance activities at the World Health Organization’s Regional Polio Laboratory in Senegal during 2013–2014. Following WHO guidelines (*7*), the laboratory received and processed stool specimens from 1,600 children with AFP from various West Africa countries. NPEV was found in isolates from most countries except Cape Verde (0/5 specimens): Gambia (8/64), Guinea-Bissau (8/49), Guinea (42/355), Mauritania (20/108), Niger (95/596), and Senegal (63/423). NPEV was initially characterized by amplification of the VP1 capsid protein coding region by using reverse transcription PCR and partial sequencing, as described (*8*). After molecular typing of all NPEV isolates, we identified 4 new EV-A71 isolates from 4 patients, 1 each from Guinea, Mauritania, Niger, and Senegal (Technical Appendix Table 1). BLAST analysis (http://www.ncbi.nlm.nih.gov/) of the partial VP1 nucleotide sequences showed that the isolates from this study shared 96%–97% nt sequence identity with those of other EV-A71 isolates deposited in GenBank.

The complete VP1 nucleotide sequences of the 4 EV-A71 isolates were determined by using reverse transcription PCR (GenBank accession nos. KT818793–KT818796; Technical Appendix Table 2). Sequences were aligned by using ClustalW (http://www.clustal.org). Phylogenetic investigation indicated that the EV-A71 strains detected in 3 of the 4 isolates (from Senegal, Guinea, and Mauritania) clustered within subgenogroup C2. The most closely related EV-A71 strains are not those previously reported from other Africa countries (Madagascar, Cameroon, Nigeria, and Central African Republic) but are strains found in isolates from France, Finland, United Kingdom, Spain, Germany and Netherlands, all isolated during 2006–2010 (Figure). The C2 clusters from Africa and Europe showed an average of 97.1% nt identity and 99.8% aa similarity. Within the Africa C2 cluster, the nucleotide and amino acid alignments displayed a substantial proportion of conserved positions: 862/891 (96.7%) and 297/297 (100%), respectively. Because most enteroviruses evolve at the rate of ≈1% nt substitutions per year in the VP1 region, the sequence divergence of ≈3% suggests that these enteroviruses had been circulating in the region for ≈3 years before they were detected. However, we cannot exclude the possibility that these isolates originated from multiple importation events from abroad, especially from countries in Europe. The fourth isolate (from Niger) clustered within genogroup E, which, before this study, included only 2 complete VP1 sequences from isolates from Central African Republic and Cameroon and an additional partial VP1 sequence from an isolate from Nigeria.

**Figure:**
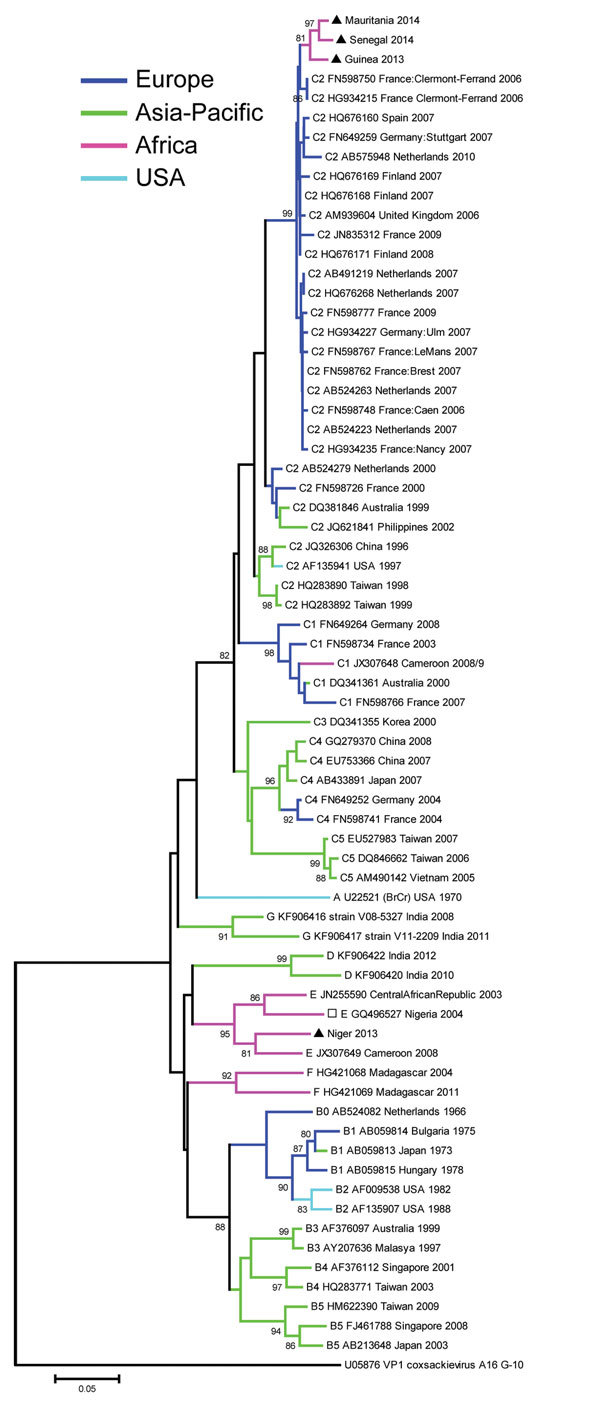
Phylogenetic tree created with the complete VP1 nucleotide sequences (891 bp in length) of enterovirus A71 isolated from 4 patients with acute flaccid paralysis in West Africa, the most similar nucleotide sequences identified by a search in GenBank by using BLAST (http://www.ncbi.nlm.nih.gov/), and a representative global set of enterovirus A71 sequences belonging to different genogroups and subgenogroups. The coxsackievirus A16 prototype G-10 sequence was introduced as the outgroup. The tree was inferred with a neighbor-joining method that used MEGA5 software (http://www.megasoftware.net/). Distances were computed by using the Kimura 2–parameter model. The robustness of the nodes was tested by using 1,000 bootstrap replications. Bootstrap support values >80 are shown in nodes. The 4 black triangles indicate the 4 strains from this study. The open square represents a partial sequence. Scale bar represents nucleotide substitutions per site. Abbreviations of virus names indicate genogroups or subgenogroups/GenBank accession number/origin/year of isolation, respectively.

Comparison of complete VP1 amino acid sequences of all EV-A71 strains considered for the phylogenetic analysis showed that 107 (36%) of 297 aa sites were variable. None of the residues found in the variable sites of the Africa strains in our study corresponded to residues previously associated with genogroup C neurovirulent phenotypes (A170V, N31D, L97R, G145E and D164E) (*9**,**10*). The Niger E isolate showed specific residues (L24M, A170T) that differed from those of other genogroup E isolates.

Our findings highlight the presence of EV-A71 with a high degree of genetic diversity in patients with AFP in West Africa. Future studies about the burden of disease, epidemiologic features, and evolution of EV-A71 in this region of Africa are needed to implement appropriate public health measures.

**Technical Appendix.** Clinical features of patients with isolates of enterovirus A71; primers and PCR conditions used for sequencing the isolates, West Africa, 2013–2014.
