# Supplementary material for: Enterovirus A71 Genogroups C and E in Children with Acute Flaccid Paralysis, West Africa
Source: Emerg Infect Dis. 2016 Apr;22(4):753–5. doi: 10.3201/eid2204.151588 (PMC4806963; doi:10.3201/eid2204.151588)
Supplement: Supplementary file 1 — Technical Appendix. Clinical features of patients with isolates of enterovirus A71; primers and PCR conditions used for sequencing the isolates, West Africa, 2013–2014. [file 15-1588-Techapp-s1.pdf]

Article DOI: http://dx.doi.org/10.3201/eid2204.151588

Enterovirus A71 Genogroups C and E in Children with Acute Flaccid Paralysis, West Africa

Technical Appendix

Technical Appendix Table 1. Clinical features of 4 patients with acute flaccid paralysis, from whose specimens EV-A71 was isolated, West Africa, 2013–2014*

	Strain GenBank accession no.			
Characteristic	KT818796	KT818795	KT818793	KT818794
Country of isolation	Niger	Guinea	Senegal	Mauritania
Region	Zinder	Labé	Thies	Assaba
Sex	M	M	F	F
Age at diagnosis, y	1.3	1.7	3	1.6
Paralysis onset date	2013/02/26	2013/04/24	2014/03/17	2014/05/06
Date of first sampling	2013/03/06	2013/04/29	2014/03/26	2014/05/22
Fever at onset of paralysis	yes	yes	yes	yes
Progressive paralysis within 3 d	yes	yes	yes	no
Asymmetric paralysis	yes	no	yes	yes
Number OPV doses	4	2	unknown	4
Last OPV vaccination date	2012/05/15	2011/10/11	unknown	2013/10/26

*AFP, acute flaccid paralysis; EV-71, enterovirus 71; OPV, oral polio vaccine.

Technical Appendix Table 2. Primers and PCR conditions used for the amplification and sequencing of the whole VP1 nucleotide sequences of EV-A71 isolates, West Africa, 2013–2014*

Name of primer	Sequence, 5′→ 3′	Location	PCR conditions
VP3_C2__Fw	AACACTCACTACAGAGCGCACG	2232–2254	94° 10 min (94°C for 1 min + 64°C for 1 min + 72°C for 2 min) × 40, 72°C for 10 min
2A_C2_Rev	GCAAGATGKCGGTTGACCACTC	3397–3376	
VP3_E_Fw	GTCATCTGGGATTTYGGGCT	2175–2194	94° 10 min (94°C 1 min + 55°C 1 min + 72°C 2 min) × 40, 72°C 10 min
2A_E_Rev	CCTTGAGCRGTAGTGGATGA	3475–3456	

*Primers for genogroup E were designed on the basis of the 2 EV-A71 sequences from genogroup E (GenBank accession nos. JN255590 and JX307649). Nucleotide positions of primers' extremities (locations) are relative to the EV-A71 prototype strain BrCr (GenBank accession No.U22521). EV-A71, enterovirus A71.
